# Functional Connectome Correlates of Laterality Preferences: Insights into Hand, Foot, and Eye Dominance across the Lifespan

**DOI:** 10.1523/ENEURO.0580-24.2025

**Published:** 2025-07-03

**Authors:** Link Tejavibulya, Corey Horien, Carolyn Fredericks, Bronte Ficek, Margaret L. Westwater, Dustin Scheinost

**Affiliations:** ^1^Interdepartmental Neuroscience Program, Yale School of Medicine, New Haven, Connecticut 06510; ^2^MD/PhD Program, Yale School of Medicine, New Haven, Connecticut 06510; ^3^Departments of Neurology, Yale School of Medicine, New Haven, Connecticut 06510; ^4^Radiology and Biomedical Imaging, Yale School of Medicine, New Haven, Connecticut 06510; ^5^Departments of Biomedical Engineering, Yale University, New Haven, Connecticut 06510; ^6^Statistics and Data Science, Yale University, New Haven, Connecticut 06510; ^7^The Child Study Center, Yale School of Medicine, New Haven, Connecticut 06510

**Keywords:** brainwide associations, eyedness, footedness, functional connectivity, handedness

## Abstract

Humans exhibit laterality preferences, with handedness being the most extensively studied. Accordingly, brain–handedness associations are well documented. However, laterality preferences extend beyond handedness to include other limbs, such as footedness and eyedness. Despite these distinctions, brain–footedness and brain–eyedness associations using resting-state functional connectomes remain largely unexplored. We utilize two large datasets, the Human Connectome Project-Development (HCP-D) and Human Connectome Project-Aging (HCP-A), to study the associations between sidedness (i.e., handedness, footedness, and eyedness) and whole-brain functional connectomes. While hand and foot preferences were correlated significantly, they explained <40% of the variance, suggesting some distinctions between measures. For both cohorts, significant associations between handedness connectivity were observed [*p* < 0.05, network-based statistics (NBS) corrected]. Notable patterns include increased connectivity for left-handedness in the posterior temporal areas and right-handedness in cerebellar regions. In contrast, significant associations between footedness and handedness connectivity were observed only in the HCP-A (*p* < 0.05, NBS corrected) and not the HCP-D. No significant associations between eyedness and connectivity were observed for either group. Finally, we compared the effect size between brain–handedness and brain–footedness associations. A greater difference was found in the HCP-D. The two cohorts primarily differed in edge distribution in the prefrontal lobe, temporal lobe, and cerebellum. Overall, in adults, brain–handedness and brain–footedness associations were similar. However, in children to adolescents, brain–handedness and brain–footedness associations diverge, suggesting a developmental shift. Characterizing sidedness associations with whole-brain connectomes may provide important insights into understanding the motor and visual systems, rehabilitation and occupational therapy, and benchmarking normative variations in the connectome.

## Significance Statement

Lateral preferences stem from functional biases in brain hemispheres. However, studies associating brain connectivity to these predominant preferences have often been oversimplified to handedness. Although approximately 90% of the population is right-handed, other lateral preferences—like footedness and eyedness—show much lower and more varied distributions. This study aims to provide a foundational understanding of how broader lateral preferences might offer a more nuanced view of the brain's functional connectivity.

## Introduction

Humans have laterality preferences, with handedness being the most studied. Not only does handedness play a role in everyday life with motor control, but it has been linked to various physical and mental health outcomes (Klar, 1999; Deep-Soboslay et al., 2010; Hirnstein and Hugdahl, 2014; Wiberg et al., 2019; Sainburg and Duff, 2006). Accordingly, brain–handedness associations are well-known. The brains of left-handed individuals are organized differently regarding language, motor, somatosensory, and the corpus callosum (Habib et al., 1991; Gut et al., 2007). Studies have shown that language (Knecht et al., 2000; Szaflarski et al., 2002; Wilberg et al., 2009; Ocklenburg et al., 2014; Esteves et al., 2021), motor (Solodkin et al., 2001; Hammond, 2002; Hatta, 2007), and somatosensory (Hoshiyama and Kakigi, 1999; Sörös et al., 1999; Jung et al., 2008) networks demonstrate robust activation and connectivity differences between left- and right-handed individuals. Recent work has shown that brain–handedness associations extend beyond localized regions of interest to widespread functional connectivity differences, affecting every canonical brain network (Faurion et al., 1999; Perlaki et al., 2013; Tejavibulya et al., 2022).

Laterality preferences extend far beyond handedness. Preferences exist for other limbs and organs, including feet (i.e., footedness) and eyes (i.e., eyedness). It is often assumed that other laterality preferences follow handedness, and, as such, they remain understudied compared with handedness (Elias et al., 1998). Nevertheless, these preferences are unique and potentially have different brain associations. For example, although 90% of the population is right-handed, this percentage drops considerably for foot and eye preference (Bourassa et al., 1996; Muraleedharan et al., 2020). Putatively, footedness predicts cognition and language better than handedness (Elias et al., 1998). Similarly, changes in ocular dominance (i.e., switch from right eye preference to left eye preference) cause neural circuit changes in the visual cortex (Sato and Stryker, 2008). Although distinct from handedness, brain–footedness and brain–eyedness associations using whole-brain functional connectomes have not been studied. Such associations may have important insights for understanding the motor and visual systems, for rehabilitation and occupational therapy, and for benchmarking normative variations in the connectome.

In this study, we utilize two large datasets, the Human Connectome Project-Development (HCP-D; Harms et al., 2018; Somerville et al., 2018) and Human Connectome Project-Aging (HCP-A; Harms et al., 2018; Bookheimer et al., 2019), to study the associations between sidedness (i.e., handedness, footedness, and eyedness) and whole-brain functional connectivity. Since brainwide associations with handedness are established (Tejavibulya et al., 2022), we utilize handedness as a comparison benchmark. We investigated behavioral correlations between sidedness measures, whole-brain associations of footedness and eyedness, differences in effect sizes between brain–handedness and brain–footedness associations, and differences between the two cohorts in these associations.

## Materials and Methods

### Datasets: Human Connectome Project-Development (HCP-D) and Human Connectome Project-Aging (HCP-A)

We analyzed sidedness and resting-state fMRI data from the Human Connectome Project-Development (HCP-D; Van Essen et al., 2012; Harms et al., 2018; Somerville et al., 2018) and the Human Connectome Project-Aging (HCP-A; Harms et al., 2018; Bookheimer et al., 2019). The HCP-D consisted of individuals aged 5–21 years, whereas the HCP-A included participants between ages 36 and 100 years. For the analyses comparing sidedness across measures, individuals were excluded if they were missing sidedness measures. These analyses include *n* = 486 individuals from the HCP-D and *n* = 724 individuals from the HCP-A. For analyses using the resting-state data, individuals were excluded if they had >0.1 mm of average frame-to-frame head motion. These analyses include *n* = 465 individuals from the HCP-D and *n* = 368 individuals from the HCP-A. Demographic information is presented in Table 1.

**Table 1.** Summary of demographics represented in the HCP-D and HCP-A

	HCP-D		HCP-A	
	Behavioral	Brain	Behavioral	Brain
Sample size	488	465	724	336
Age	16.16 ± 3.12	15.12 ± 3.77	60.35 ± 15.73	56.34 ± 13.48
	Range: 11–21.92	Range: 8.08–21.92	Range: 36–100	Range: 36–100
Sex (M/F)	255/233	252/213	406/318	195/173
Handedness (normalized)	63.96 ± 43.98	60.91 ± 41.90	50.37 ± 37.27	63.69 ± 53.25
	IQR = 55.56–88.89	IQR = 55.56–88.89	IQR = 50–75	IQR = 61.11–100
Footedness	59.11 ± 56.99	58.25 ± 57.88	66.16 ± 54.41	64.48 ± 55.56
	IQR = 50–100	IQR = 50–100	IQR = 50–100	IQR = 50–100
Eyedness	19.57 ± 73.50	18.98 ± 73.44	24.55 ± 70.15	26.49 ± 70.06
	IQR = −50 to 100	IQR = −50 to 100	IQR = 0–100	IQR = −12.5 to 100

### Behavioral measures of sidedness

Handedness was determined from individuals’ self-reported preferences for their dominant hand using nine items from the Edinburgh Handedness Inventory (Oldfield, 1971). Participants were asked which hand they preferred to write, throw a ball, use a spoon, brush their teeth, use an upper hand when using a broom, light a match, open a box, use scissors, and use a knife (without a fork). Footedness was determined from individuals’ self-reported preferences for their dominant foot to kick. Eyedness was determined from individuals’ self-reported preferences for their dominant eye to keep open when looking through a telescope. Individuals responded using a five-point Likert scale, ranging from 1 “always left” to 5 “always right,” to indicate their preference for each. All measures were kept as ordinal data rather than binarizing the data to provide dimensional measures of sidedness. The counts of each response are shown in Tables 2 and 3.

**Table 2.** Table of handedness, footedness, and eyedness concordance for HCP-D

	Always left	Left	Indifferent	Right	Always right
Writing	66	1	0	18	567
Throwing	41	11	20	103	477
Scissors	45	8	22	61	516
Toothbrush	51	12	50	87	451
Knife (no fork)	29	18	28	73	340
Spoon	57	11	31	75	477
Broom	73	63	96	74	182
Match	26	10	24	57	371
Box	28	16	123	86	235
Foot	30	16	52	127	263
Eye	79	66	93	85	165

**Table 3.** Table of handedness, footedness, and eyedness concordance for HCP-A

	Always left	Left	Indifferent	Right	Always right
Writing	81	3	0	18	623
Throwing	50	12	24	87	552
Scissors	45	9	20	71	580
Toothbrush	72	14	39	77	523
Knife (no fork)	55	16	32	84	538
Spoon	62	17	30	101	515
Broom	84	65	109	97	370
Match	59	11	18	72	564
Box	49	27	134	134	381
Foot	39	19	58	161	447
Eye	96	85	159	135	249

### Imaging data

All HCP-D and HCP-A neuroimaging was conducted on a 3T Siemens Prisma scanners (Siemens, Erlangen, Germany) using the Siemens 32-channel Prisma head coil. The resting-state scans are acquired with a 2D multiband gradient-recalled echoplanar imaging sequence (multiband factor, 8; TR, 800 ms; TE, 37 ms; flip angle, 52°; 2.0 mm isotropic voxels; and 72 slices). T1w scans are acquired with a multiecho MPRAGE sequence (0.8 mm isotropic voxels; sagittal FOV, 256 × 240 × 166 mm; matrix size, 320 × 300 × 208 slices; TR/TI, 2,500/1,000; TE, 1.8/3.6/5.4/7.2 ms; flip angle, 8°).

### Preprocessing and generating connectomes

Both the HCP-D and HCP-A datasets were analyzed with identical processing pipelines. T1w scans were first skull-stripped using an optimized version of the FMRIB's Software Library (Smith et al., 2004) pipeline (Lutkenhoff et al., 2014). Functional images were motion-corrected using SPM12. All further analyses were performed using BioImage Suite (Joshi et al., 2011). Several covariates of no interest were regressed from the data, including linear and quadratic drifts, mean cerebral spinal fluid signal, mean white matter signal, and mean gray matter signal. For additional control of possible motion-related confounds, a 24-parameter motion model (including six rigid-body motion parameters, six temporal derivatives, and these terms squared) was regressed from the data. The data were temporally smoothed with a Gaussian filter (approximate cutoff frequency, 0.12 Hz).

Nodes were defined using the Shen 268-node brain atlas (Shen et al., 2013), which includes the cortex, subcortex, and cerebellum. The atlas was warped from MNI space into single-subject space via a series of linear and nonlinear transformations calculated using a previously validated algorithm (Scheinost et al., 2017), implemented in BioImage Suite. Resting-state connectivity was calculated as the mean time courses for each of the 268 nodes (i.e., averaging the time courses of all constituent voxels). Node-by-node pairwise correlations were computed, and Pearson’s correlation coefficients were Fisher *z*-transformed to yield symmetric 268 × 268 connectivity matrices, in which each matrix element represents the connectivity strength between two individual nodes (i.e., “edge”).

### Statistical analyses

To investigate the associations between handedness, footedness, and eyedness, Spearman's correlations (*ρ*) were calculated between pairs of sidedness measures and subsequently squared to obtain the variance explained. We used *Z*-tests and Steiger's *z*-test for dependent correlations to compare effect sizes (Steiger, 1980; Lee and Preacher, 2013) between associations and groups.

We separately assessed edge-wise Spearman's correlations with the handedness, footedness, and eyedness scores for the HCP-D and HCP-A datasets to investigate brain–handedness, brain–footedness, and brain–eyedness associations. We used network-based statistics (NBS; Zalesky et al., 2010) to identify components where functional connectivity was significantly associated with handedness while controlling for the familywise error rate. We used a component-determining threshold *z* = 1.96, two tails, and *K* = 5,000 permutations. NBS is analogous to cluster-based correction and solves the statistical problem of massively multiple comparisons in a whole-brain connectivity analysis. In NBS, using the correlation between sidedness measures and connectivity, the largest fully connected network of suprathreshold edges, or “component,” is identified, and its extent is defined as the number of edges it comprises. Finally, these calculations are repeated for 5,000 iterations, in which subjects’ group assignments are randomly permuted to create a null distribution for the expected component size due to chance. NBS results are shown at *p* < 0.05, corrected for multiple comparisons.

We examined the overlap of significant edges from the NBS analyses to investigate the convergence of connectivity profiles across sidedness measures. Significance was determined with the hypergeometric cumulative density function (Rosenberg et al., 2016), which returns the probability of drawing up to *x* of *K* possible items in *n* drawings without replacement from an *M*-item population. In other words, this approach calculates the probability of finding the number of overlapping from two independent analyses by chance. Given many possible edges (>35,000), finding overlapping edges when randomly sampling two sets of ∼500 edges is rare. This was implemented in Matlab as: *p* = 1-hygecdf (*x*, *M*, *K*, *n*), where *x* is the number of overlapping edges, *K* is the number of connections in the HCP-D, *n* is the number of connections in the HCP-A, and *M* is the total number of edges in the matrix (35,778).

As thresholding results based on significance can modulate observed group differences (Taylor et al., 2023), we also examined the effect sizes for brain–handedness, brain–footedness, and brain–eyedness associations for the HCP-D and the HCP-A datasets without thresholding for significance. We quantified the effect size using Cohen's *d*. To investigate if edges strongly associated with handedness were strongly associated with footedness or eyedness, we correlated effect sizes for all 35,778 edges. We used *Z*-tests to compare these correlations across groups.

Finally, we compared brain–handedness and brain–footedness associations using edge-wise Steiger's *z*-test (Steiger, 1980; Lee and Preacher, 2013) to account for the correlation between handedness and footedness. Chi-squared tests were used to quantify significant differences in edge distributions between the HCP-A and HCP-D.

## Results

### Behavioral association between handedness, footedness, and eyedness

**Figure 1.**
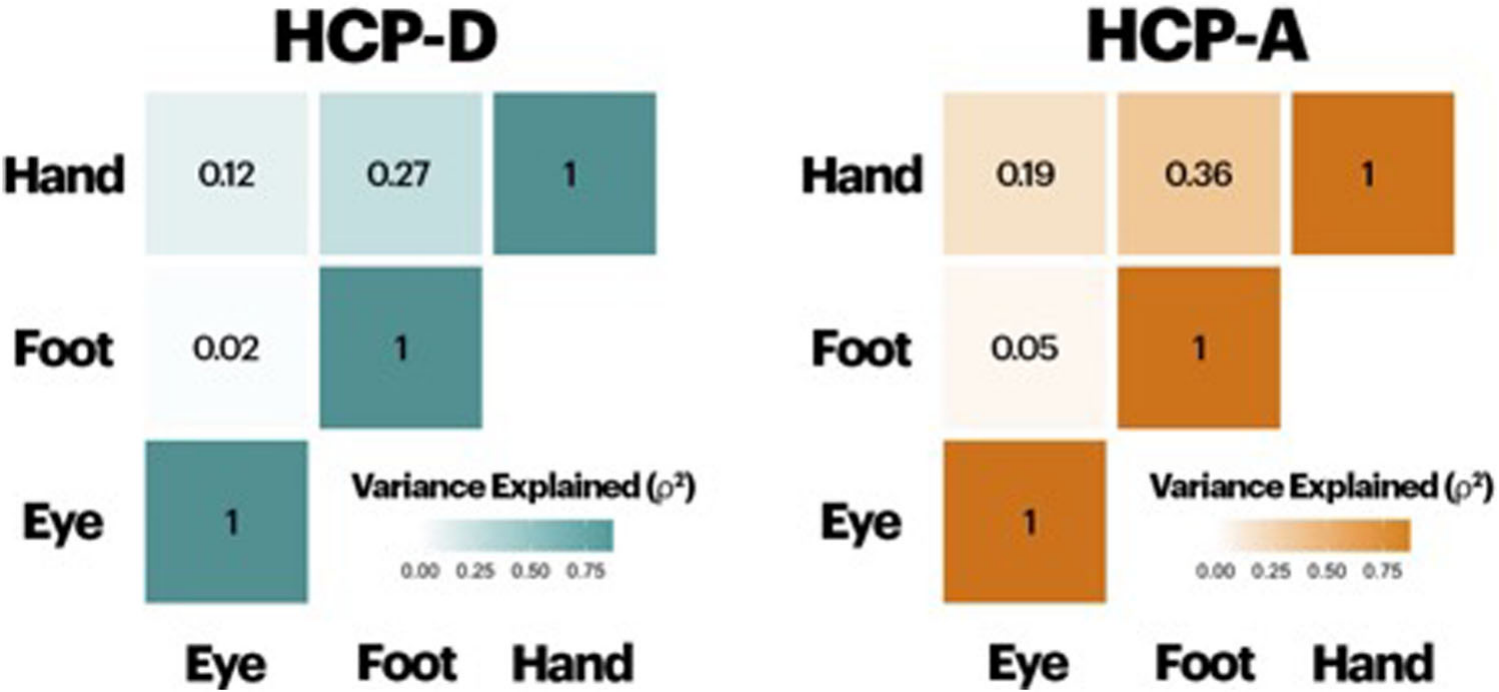
Behavioral correlations between sidedness measures. While handedness and footedness were correlated significantly, they explained <40% of the variance of the other one, suggesting some distinctions between the measures. Correlation heatmaps are shown as variance explained (*r*^2^) for each pair of sidedness measures in both cohorts.

Spearman's correlations (*ρ*) between pairs of sidedness (e.g., handedness, footedness, and eyedness) are shown in Figure 1. In both cohorts, all associations were significant (*r*'s > 0.14, *p*'s < 0.05, corrected). However, the correlation between handedness and footedness and between handedness and eyedness were significantly greater than the correlation between footedness and eyedness (*z*'s > 4.58, *p*'s < 0.001) in both cohorts. Significantly stronger associations were observed in the HCP-A compared with the HCP-D (*z*'s > 1.65, *p*'s < 0.05). Overall, while handedness and footedness were correlated significantly, they explained <40% of the variance of the other one, suggesting some distinctions between the measures.

### Functional connectivity associations with handedness, footedness, and eyedness

**Figure 2.**
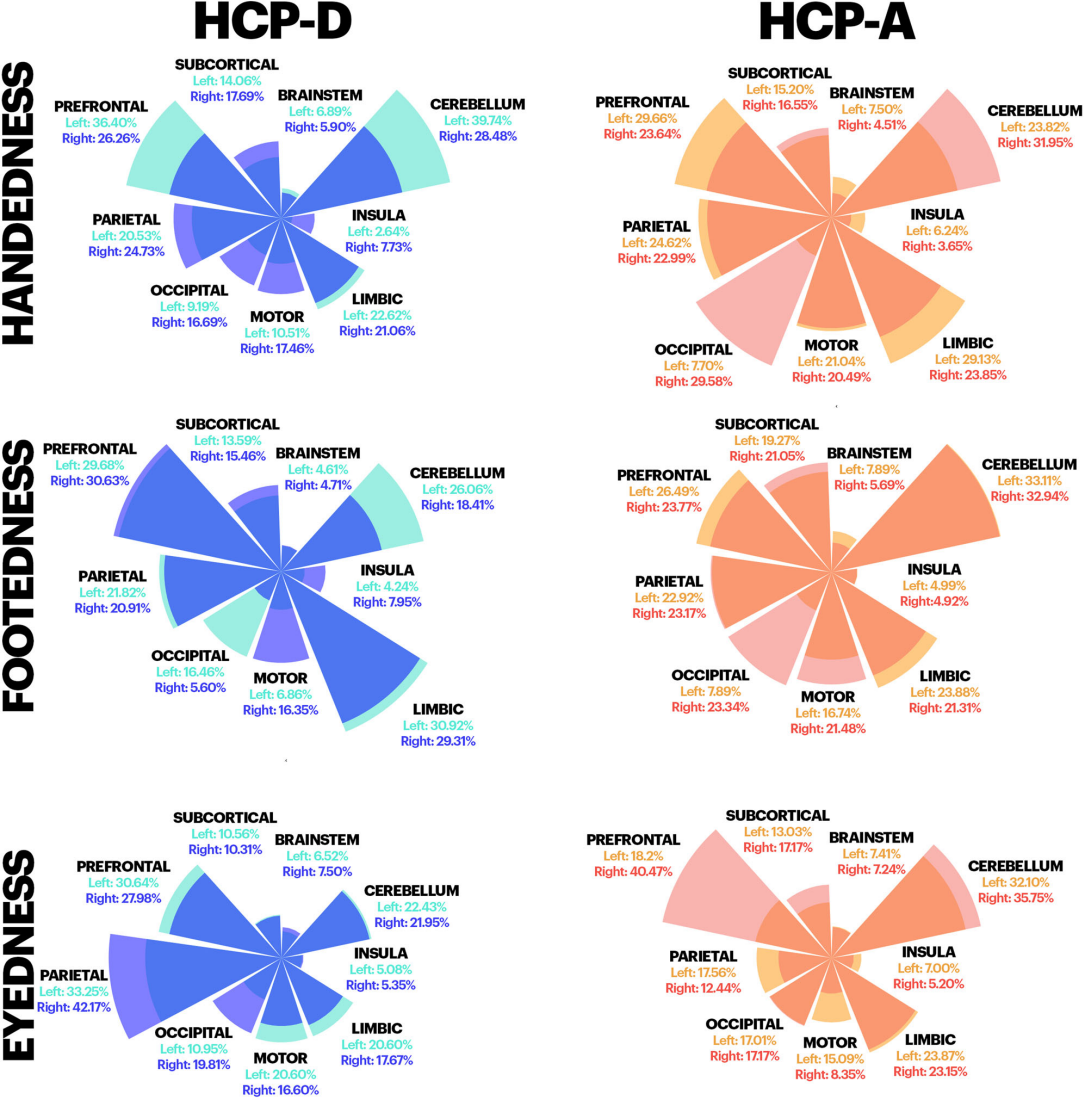
Edges significantly differ between left- and right-sided individuals as calculated by network-based statistics. Circular bar plots showing the percent of significant edges belonging to each canonical network for each sidedness measure (rows) as separated by datasets (columns). Each circular bar plot depicts percentages for left- (cyan for HCP-D and salmon for HCP-A) and right-sided edges (blue for HCP-D and red for HCP-A). Handedness yielded a significance of *p* < 0.001 for both the HCP-A and the HCP-D, whereas footedness only yielded a significance of *p* < 0.001 for the HCP-A. The remainder of the results show no significant differences.

For both cohorts, significant associations between handedness and connectivity were observed (*p* < 0.05, NBS correction). These associations were widespread, with edges in every functional network and lobe, mirroring our previous results in an independent cohort (Tejavibulya et al., 2022). In the HCP-A, 1,507 edges were positively associated with left-handedness, and 1,396 were positively associated with greater right-handedness. In the HCP-D, 1,437 edges were positively associated with left-handedness, and 1,306 were positively associated with greater right-handedness. Notable patterns include prominent representations in prefrontal and cerebellar regions for both left- and right-handedness in both datasets. In the HCP-A, right-handedness was highly represented in the occipital network attributing to 29.58% of edges, whereas left-handedness was shown to be represented in the limbic network at 29.13% of edges (Fig. 2, top row, orange circular plot). In contrast, edges attributing to differences in handedness in the HCP-D were mostly confined within the cerebellar and prefrontal networks (Fig. 2, top row, blue circular plot). Overall, detected edges significantly overlapped between the two cohorts (*p* < 0.001), suggesting connectivity profiles of handedness were consistent across the HCP-A and HCP-D.

In contrast, significant associations between footedness and connectivity were observed only in the HCP-A (*p* < 0.05, NBS correction). In the HCP-A, 1,344 edges were positively associated with greater left-footedness, and 1,178 were positively associated with greater right-footedness. Like handedness, these associations were widespread, with edges in every functional network and lobe significantly overlapping with the connectivity profiles for handedness (*p* < 0.001). More specifically in the HCP-A, significant edges associated with right-footedness were distributed across multiple networks at 29.58, 20.49, and 21.31% for the occipital, motor, and limbic networks in right-handedness, respectively, while with left-handedness significant edges were more confined within the parietal and prefrontal networks at 24.62 and 29.66%, respectively. Although NBS results did not yield significance in the HCP-D, the limbic networks were associated with footedness differences with 30.92 and 29.31% of edges in left- and right-footedness, respectively, connected either within or between the limbic network. The edges associated with greater left-footedness and left-handedness in the HCP-A significantly overlapped (overlapping edges, 605; *p* < 0.001). Similarly, the edges associated with greater right-footedness and right-handedness in the HCP-A significantly overlapped (overlapping edges, 505; *p* < 0.001). Overall, handedness and footedness exhibited similar significant associations with functional connectivity only in HCP-A.

No significant associations between eyedness and connectivity were observed for either group; however, results are shown here for consistency.

### Comparisons of effect sizes

**Figure 3.**
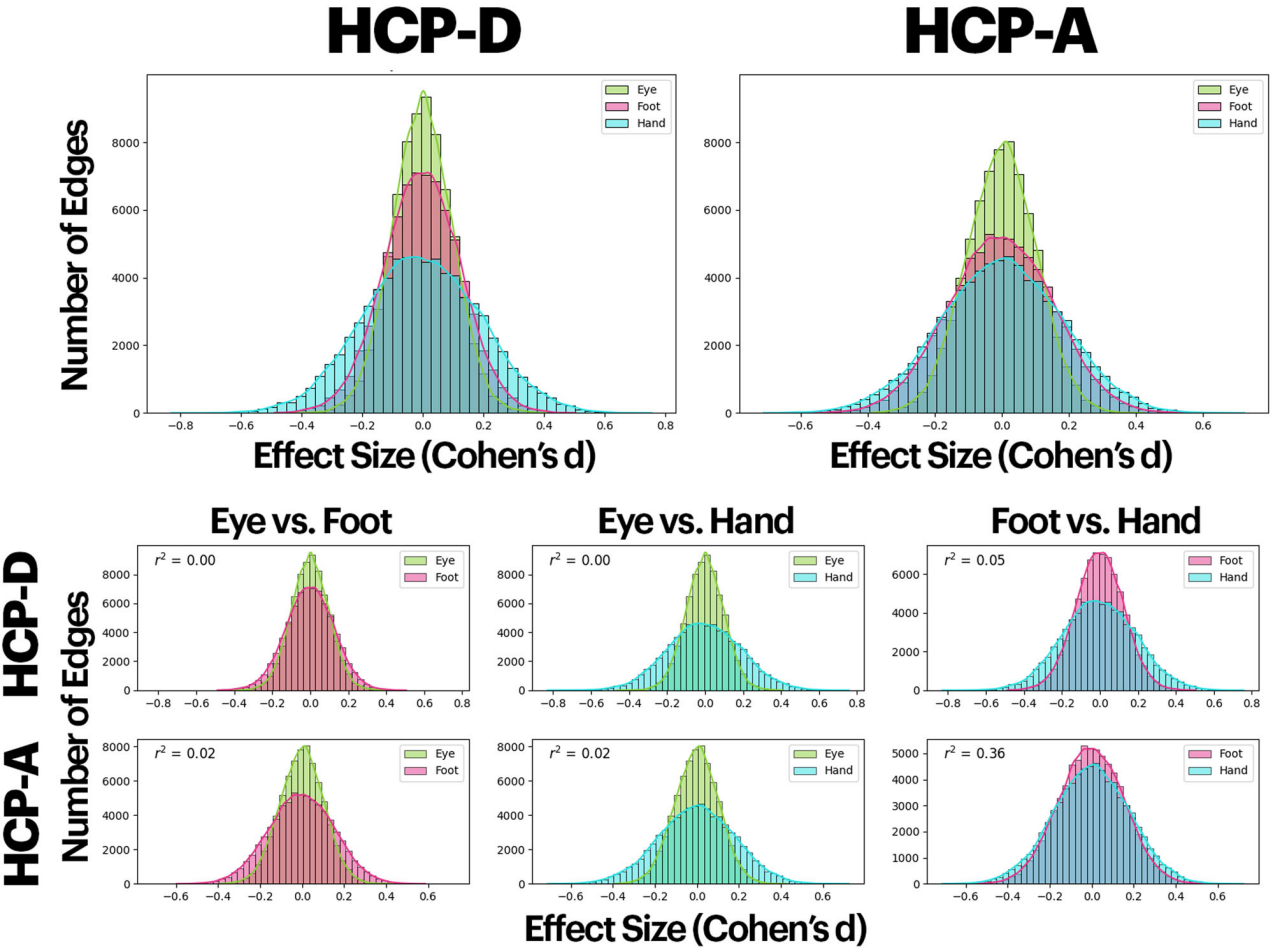
Effect size quantifications and correlations for all edges in each sidedness measure. Top, Effect sizes (Cohen's *d*) for all 35,778 were calculated and plotted on a histogram for each sidedness measure and cohort separately. A wider histogram indicates that more edges show a larger effect size. Bottom, Pairs of sidedness measures from the top were further compared individually and accompanied with calculations of covariances for the HCP-D and the HCP-A, separately.

The distributions of effect sizes across all edges (regardless of significance) for connectivity associations with handedness, footedness, and eyedness in the HCP-D and HCP-A, independently, are shown in Figure 3. Aligned with Figure 2, brain–eyedness associations exhibited the narrowest effect size distributions (i.e., smaller effect sizes between connectivity and eyedness), followed by footedness and handedness. Across all three measures of sidedness, the distributions of effect sizes in the HCP-D were narrower than that of the HCP-A, indicating that effect sizes were lower for all measures in the younger cohort. Visually, while the effect size distributions were similar for handedness and footedness between the HCP-A and HCP-D, the effect size distribution of footedness for the HCP-D was much narrower than that for the HCP-A. Overall, the effect sizes for footedness in the HCP-D are smaller and closer to that of eyedness. In contrast, the effect sizes for footedness in the HCP-A are much larger and more closely resemble handedness.

Additionally, in the HCP-A, edges strongly associated with handedness were also strongly associated with footedness (Fig. 3; *r* = 0.60, *p* < 0.001), suggesting that similar edges are involved in handedness and footedness. This correlation was significantly weaker in the HCP-D (Fig. 3; HCP-D, *r* = 0.22, *p* < 0.001; *Z* = 6.704, *p* < 0.001). In both cohorts, edges with strong eyedness associations were independent of those with strong associations with handedness or footedness.

### Comparison between handedness and footedness associations with connectivity

**Figure 4.**
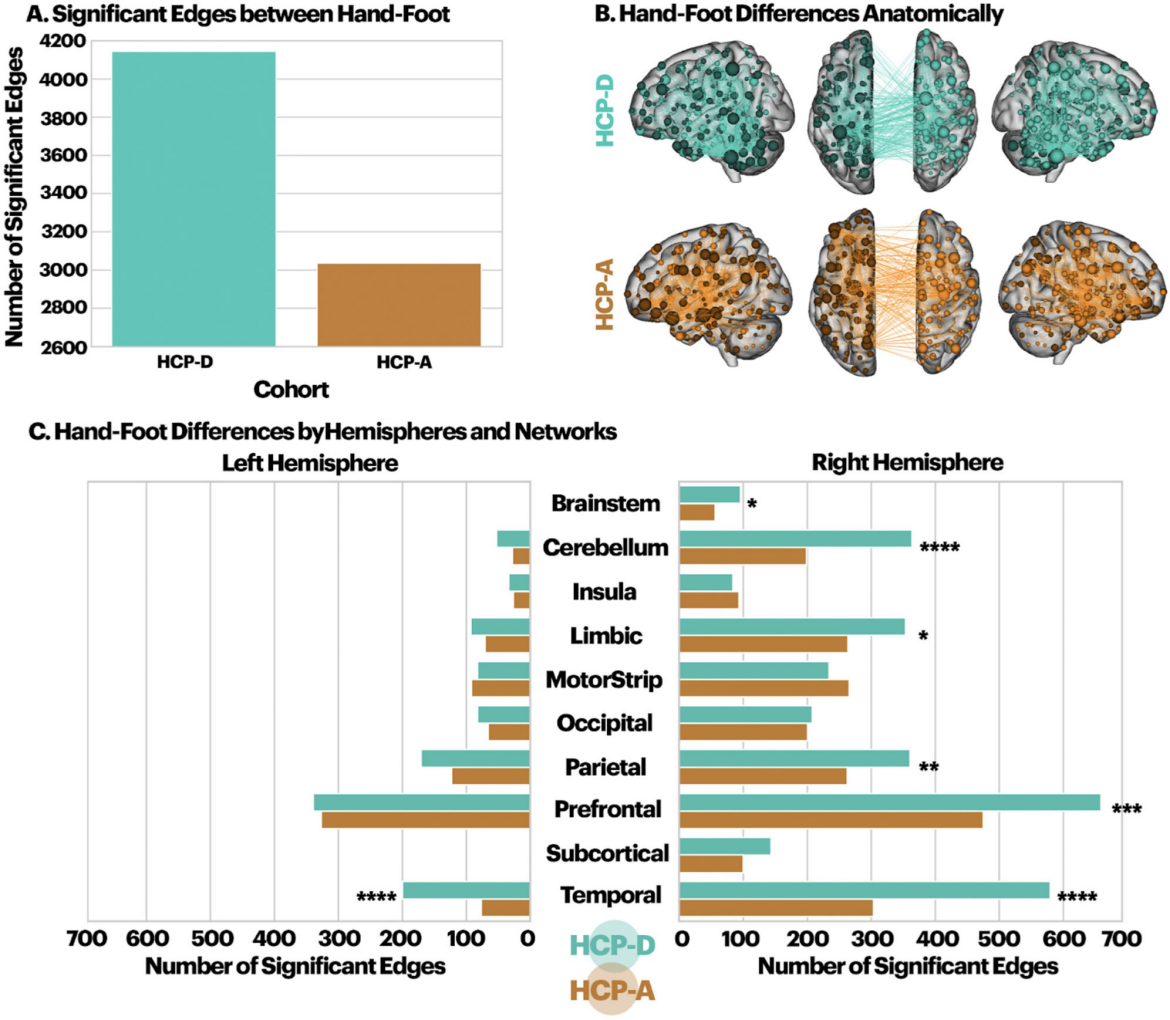
Edges with significantly different effect sizes in handedness and footedness for each cohort separately. ***A***, The number of edges with significantly different effect sizes for brain–handedness and brain–footedness associations, separated by cohort. As expected, the number of edges was significantly greater in the HCP-D than in the HCP-A (*x*^2^ = 8.1, *p* < 0.001). ***B***, These edges were located throughout the brain. Results for all visualizations were thresholded as follows to aid with interpretability (70 for HCP-D and 50 for HCP-A). ***C***, Edges with significantly different effect sizes for brain–handedness and brain–footedness associations split by hemispheres and networks. * indicates networks where the HCP-D exhibited significantly more edges that differed in effect size between handedness and footedness.

When comparing brain–handedness and brain–footedness associations, 3,035 edges exhibited significantly different effect sizes in the HCP-A, and 4,143 edges exhibited significantly different effect sizes in the HCP-D. As expected, the number of edges was significantly greater in the HCP-D than in the HCP-A (Fig. 4*A*; *x*^2^ = 8.1, *p* < 0.001), suggesting a mismatch of handedness and footedness. Surprisingly, for both cohorts, most of these edges were located in the right hemisphere (*χ*^2^ = 870.26, *p* < 0.0001). The two cohorts primarily differed in edge distribution in the prefrontal lobe, temporal lobe, and cerebellum (Fig. 4*B*,*C*).

## Discussion

We investigated how lateral preferences—extending beyond handedness preferences—associate with the brain's functional organization across two distinct cohorts and generations using data from the HCP-Development and HCP-Aging datasets. Behavioral associations between handedness, footedness, and eyedness showed weaker correlations in the younger cohort (HCP-D) than in the older cohort (HCP-A). Footedness and handedness associations strengthened in the older cohorts, while eyedness exhibited no associations between the other two measures. For both cohorts, significant associations between handedness and connectivity were observed. Notable patterns include increased connectivity for left-handedness in the posterior temporal areas and right-handedness in cerebellar regions. In contrast, significant associations between footedness and connectivity were observed only in the HCP-A. These connectivity patterns were similar to the handedness results. No significant associations between eyedness and connectivity were observed for either group. Finally, we compared the effect size between brain–handedness and brain–footedness associations. A greater difference was found in the HCP-D. The two cohorts primarily differed in edge distribution in the prefrontal lobe, temporal lobe, and cerebellum. Overall, in adults, brain–handedness and brain–footedness associations were similar. However, in children to adolescents, brain–handedness and brain–footedness associations diverge, suggesting a developmental shift. Characterizing sidedness associations with whole-brain connectomes provides a starting point for benchmarking normative variations.

Across analyses, the cerebellum and prefrontal cortex consistently showed differential connectivity patterns associated with handedness and footedness. The cerebellum is known to develop substantially postnatally (Friede, 1973), and both the cerebellum and the prefrontal cortex are heavily influenced by environmental factors (Rossi et al., 1997; Angelucci et al., 2009; Hodel, 2018). Previous studies have highlighted the cerebellum involvement in identifying left- and right-handed groups (Tejavibulya et al., 2022) and fine motor development (Kusano et al., 2021; Ashida et al., 2022; Saadon-Grosman et al., 2022). Notably, these regions extend beyond regions classically associated with laterality preferences, like language ( Knecht et al., 2000; Wiberg et al., 2019), motor (Dassonville et al., 1997; Solodkin et al., 2001; Klöppel et al., 2010), and somatosensory areas (Sörös et al., 1999; Jung et al., 2008).

Most individuals have two hands, feet, and eyes, allowing laterality preferences to occur naturally. Even so, whether we use our left or right side differently for a particular activity depends on many factors (Coren, 1993). We can simultaneously use our hands for different purposes, contributing to how strongly we prefer our left or right hands for a specific activity (Provins and Magliaro, 1993). In other words, an individual who writes with their right hand may not necessarily throw with their right hand. Similarly, most humans have uneven eyesight (Ehrenstein et al., 2005; Dupierrix et al., 2014). Purely studying handedness provides a biased perspective of laterality preferences, as proportions of right-handedness are far more polarizing than other measures of laterality (Germono, 1965; Tran et al., 2014). These variations in how an individual balances left- or right-handed tendencies across different measures are often overlooked in handedness research despite studies indicating notable differences (Good et al., 2001; Sun and Walsh, 2006; Gut et al., 2007). Previous studies have shown how and when handedness is measured greatly affects neuroscience studies on brain–handedness associations (Tejavibulya et al., 2024). Thus, genuinely capturing how lateral preferences shape the brain's functional organization requires assessments beyond handedness. Though our findings are only based on one measure of body laterality, further research using other laterality measures will be needed to confirm our results.

Despite recent advancements, left-handed individuals remain excluded mainly from human neuroimaging studies. Research has yet to determine effective methods for controlling these differences in brain function and when these differences matter to the question. Nevertheless, this large-scale exclusion is counter to broader inclusive best practices in neuroimaging (Ricard et al., 2023; Wu et al., 2024). Studies like ours that characterize the associations between handedness, footedness, eyedness, and other laterality preferences and brain function are the first step in better involving left-handed individuals in neuroimaging studies.

We showed that footedness connectivity profiles converge onto that of handedness for the older but not the younger cohort. Overall, an overlooked aspect of studies on lateral preferences in the brain is the role of developmental trajectories and critical periods (Gabbard and Iteya, 1996). The development trajectories of laterality preferences occur on distinct timelines for different body parts (Fig. 3). Speculatively, these results may offer insights into potential comorbidities between handedness and psychiatric conditions (Logue et al., 2015; Mallet et al., 2022; Mundorf et al., 2024). Adolescence is a sensitive and critical period in brain maturation, during which the risk for mental illness is significantly heightened (Chambers et al., 2003; Casey and Jones, 2010; Nixon and McClain, 2010; Casey et al., 2011; Reynolds et al., 2019). Similarly, our results suggest that it may also be a critical period for laterality preferences and their associations in the brain. Further investigation into the associations between laterality preferences, mental health, and brain development may be warranted.

Our study has several notable strengths. We investigated how different measures of body laterality, beyond handedness, differentially affect the brain's functional organization. We also used large cohorts with harmonized imaging protocols and sidedness measures. Nevertheless, our study has several limitations. One limitation of our work is the precision of measures of footedness and eyedness. In contrast to handedness, which was summed over several questions, they were reduced to a single preference question. In particular, for eyedness, participants were asked which eye individuals prefer to remain open if one were to look through a telescope. While it was the standard measure in two human connectome projects, it may be a poor measure of eyedness. Other measures of eyedness will likely give strong associations with the connectome. Thus, while we failed to show a significant eyedness association with functional connectivity, we cannot conclude for certain that brain–eyedness associations do not exist. While the brain–footedness differences between cohorts suggest a development change, we lack longitudinal data over childhood to fully elucidate potential critical periods. Many studies over different generations are likely needed to map out generational effects fully. Longitudinal datasets [such as the Adolescent Brain Cognitive Development Study (Casey et al., 2018)] may represent a starting point for further investigations.

Lateral preferences are associated with the brain's functional organization. However, studies associating brain connectivity to these predominant preferences have often been oversimplified to handedness. Although approximately 90% of the population is right-handed, other lateral preferences—like footedness and eyedness—show much lower and more varied distributions. Our study provides a broader view of lateral preferences and functional connectivity, offering a more nuanced view. Characterizing sidedness associations with whole-brain connectomes provides a starting point for understanding how and when handedness, footedness, and eyedness need to be accounted for in fMRI analyses.

## Synthesis

Reviewing Editor: Christine Portfors, Washington State University Decisions are customarily a result of the Reviewing Editor and the peer reviewers coming together and discussing their recommendations until a consensus is reached. When revisions are invited, a fact-based synthesis statement explaining their decision and outlining what is needed to prepare a revision will be listed below. The following reviewer(s) agreed to reveal their identity: NONE. Note: If this manuscript was transferred from JNeurosci and a decision was made to accept the manuscript without peer review, a brief statement to this effect will instead be what is listed below.

The reviewers agree that this is an interesting paper. The writing is clear. Some clarifications in the methods and results would be helpful.

The premise of the paper is very interesting and seeks to fill a gap in the field regarding the correlation between handedness, footedness, eyedness, and those individuals' connectomes. The introduction appears to be well researched and well cited. The discussion is also thoughtful. The overall writing of the paper was easy to read. Items that need addressing are below, regarding methods and results:

1. Please clarify some demographic data (is possible) of the HCP-A data set. In the HCP-A group, did you exclude any individuals that had suffered traumatic brain injuries, for example, or any individuals who were displaying neurodegenerative or cognitive impairments? Essentially ,did you take into account medical histories? Additionally, the age range of that data set is 36-100 years. Would it have been worth subdividing those age ranges into smaller subgroups? If not, why? Also, I assumed but it was not specifically stated that the behavioral measure of sidedness was collected from subjects whose data was used in HCP-D and HCP-A.

2. Figure 2 - I found this figure particularly confusing to understand. It was unclear to me how the figure is showing that edges differ between left and right-sided individuals. I think the confusion stems from the fact that you seemingly overlaid right sided dominant (purple) with the left sided dominant (green) on a single brain image for both groups. It is just hard to see. Also, would there be a way to quantify these edge differences and represent that in a graphical way? I also found it really confusing that the eyedness and one of the footedness images are blurred out, or gray scaled. If you are obscuring them, why include them? I do think they should be included. Finally, there is a typo in the figure legend itself - you indicate both left- and right-sided groups as represented by purple.

3. Figure 3 - The graphs should be bigger, and the coloring choices make it hard to distinguish between the foot and eye groups.

Figure 4- I had no problems with this figure and I thought it was the best laid out and easiest to follow of all of them.

## Author Response

The reviewers agree that this is an interesting paper. The writing is clear. Some clarifications in the methods and results would be helpful.

The premise of the paper is very interesting and seeks to fill a gap in the field regarding the correlation between handedness, footedness, eyedness, and those individuals' connectomes. The introduction appears to be well researched and well cited. The discussion is also thoughtful. The overall writing of the paper was easy to read. Items that need addressing are below, regarding methods and results:

1. Please clarify some demographic data (is possible) of the HCP-A data set. In the HCP-A group, did you exclude any individuals that had suffered traumatic brain injuries, for example, or any individuals who were displaying neurodegenerative or cognitive impairments? Essentially ,did you take into account medical histories? No medical history was taken into account in the HCP-A dataset. The only exclusion criteria involved were with missing data and excessive head motion in the scanner.

Analyses were performed to control for age and sex, however.

Additionally, the age range of that data set is 36-100 years. Would it have been worth subdividing those age ranges into smaller subgroups? If not, why? While we attempted to subdivide 36-100 into finger grain groups, we were limited by the amount of data that would have been possible to fully examine these subgroups. Because the data were also skewed, we would have had many more subjects in certain bins than others where some bins were limited to as little as 2 or 3 subjects.

Also, I assumed but it was not specifically stated that the behavioral measure of sidedness was collected from subjects whose data was used in HCP-D and HCP-A.

That is correct.

2. Figure 2 - I found this figure particularly confusing to understand. It was unclear to me how the figure is showing that edges differ between left and right-sided individuals. I think the confusion stems from the fact that you seemingly overlaid right sided dominant (purple) with the left sided dominant (green) on a single brain image for both groups. It is just hard to see. Also, would there be a way to quantify these edge differences and represent that in a graphical way? I also found it really confusing that the eyedness and one of the footedness images are blurred out, or gray scaled. If you are obscuring them, why include them? I do think they should be included. Finally, there is a typo in the figure legend itself - you indicate both left- and right-sided groups as represented by purple.

We have changed Fig. 2 to be represented in a graphical way demonstrated by percent significant edges by networks as shown below. We have also removed the occlusions for insignificant results and have only noted this in the caption.

These changes have been reflected in lines 548-553:

Fig. 2: Edges significantly differ between left- and right-sided individuals as calculated by Network-Based Statistics. Circular bar plots showing percent of significant edges belonging to each canonical network for each sidedness measure (rows) as separated by datasets (columns). Each circular bar plot depicts percentages for left- (cyan for HCP-D and salmon for HCP-A) and right-sided edges (blue for HCP-D and red for HCP-A). Handedness yielded significance of p < 0.001 for both the HCP-A and the HCP-D, whereas footedness only yielded significance of p < 0.001 for the HCP-A. The remainder of the results show no significant differences.

Additionally, the main text has been updated to reflect these changes as follows in lines 197-203for handedness:

Notable patterns include prominent representations in prefrontal and cerebellar regions for both left- and right-handedness in both datasets. In the HCP-A, right-handedness was highly represented in the occipital network attributing to 29.58% of edges, whereas left-handedness was shown to be represented in the limbic network at 29.13% of edges (Fig. 2: top row, orange circular plot). In contrast, edges attributing to differences in handedness in the HCP-D were mostly confined within the cerebellar and prefrontal networks (Fig. 2: top row, blue circular plot).

For footedness in lines 210-217:

More specifically in the HCP-A, significant edges associated with right-footedness were distributed across multiple networks at 29.58%, 20.49%, and 21.31% for the occipital, motor, and limbic networks in right-handedness, respectively, while with left-handedness significant edges were more confined within the parietal and prefrontal networks at 24.62% and 29.66%, respectively. Although NBS results did not yield significance in the HCP-D, the limbic networks were associated with footedness differences with 30.92% and 29.31% of edges in left- and right-footedness, respectively, connected either within or between the limbic network.

For eyedness in lines 223-224:

No significant associations between eyedness and connectivity were observed for either group, however results are shown here for consistency.

3. Figure 3 - The graphs should be bigger, and the coloring choices make it hard to distinguish between the foot and eye groups.

These graphs have been modified to make the colors more distinguishable. Additionally, these plots have also been split up by pairs of sidedness measures to visualize differences between specific pairs with covariances accompanying each graph.

These changes have been reflected in lines 555-559:

Fig. 3: Effect size quantifications and correlations for all edges in each sidedness measure. (Top) Effect sizes (Cohen's d) for all 35,778 were calculated and plotted on a histogram for each sidedness measure and cohort separately. A wider histogram indicates that more edges show a larger effect size. (Bottom) Pairs of sidedness measures from top were further compared individually and accompanied with calculations of covariances for the HCP-D and the HCP-A, separately.

Figure 4- I had no problems with this figure and I thought it was the best laid out and easiest to follow of all of them.
